# Longitudinal Speech Outcome at 5 and 10 Years in UCLP: Influence of Speech Therapy and Secondary Velopharyngeal Surgery

**DOI:** 10.1177/10556656231225575

**Published:** 2024-02-26

**Authors:** C Persson, J Davies, C Havstam, H Søgaard, M Bowden, M Boers, JB Nielsen, S Alaluusua, I Lundeborg Hammarström, BK Emborg, A Sand, A Lohmander

**Affiliations:** 1 Institute of Neuroscience and Physiology, Department of Health and Rehabilitation, Speech and Language Pathology Unit, Sahlgrenska Academy, University of Gothenburg, Gothenburg, Sweden; 2 Department of Otorhinolaryngology, Region Västra Götaland, Sahlgrenska University Hospital, Gothenburg, Sweden; 3 Greater Manchester Cleft Unit – part of North West of England, North Wales and Isle of Man Cleft Network, Manchester Universities NHS Foundation Trust, Manchester, UK; 4 Copenhagen Cleft Palate Centre, University Hospital of Copenhagen, Copenhagen, Denmark; 5 Cleft Palate Centre, Aarhus, Denmark; 6 Cleft palate and Craniofacial Center, Department of Plastic Surgery, Helsinki University Hospital, Helsinki, Finland; 7 Speech and Language Pathology, BKV, Linköping University, Linköping, Sweden; 8 Division of Speech and Language Pathology, CLINTEC, Karolinska Institutet, Stockholm, Sweden; 9 Medical Unit Speech and Language Pathology, Karolinska University Hospital, Stockholm, Sweden

**Keywords:** cleft lip and palate, speech production, speech therapy, pharyngeal flap

## Abstract

**Objective:**

To investigate speech development of children aged 5 and 10 years with repaired unilateral cleft lip and palate (UCLP) and identify speech characteristics when speech proficiency is not at ‘peer level’ at 10 years. Estimate how the number of speech therapy visits are related to speech proficiency at 10 years, and what factors are predictive of whether a child's speech proficiency at 10 years is at ‘peer level’ or not.

**Design:**

Longitudinal complete datasets from the Scandcleft project

**Participants:**

320 children from nine cleft palate teams in five countries, operated on with one out of four surgical methods.

**Interventions:**

Secondary velopharyngeal surgery (VP-surgery) and number of speech therapy visits (ST-visits), a proxy for speech intervention.

**Main Outcome Measures:**

‘Peer level’ of percentage of consonants correct (PCC, > 91%) and the composite score of velopharyngeal competence (VPC-Sum, 0–1).

**Results:**

Speech proficiency improved, with only 23% of the participants at ‘peer level’ at 5 years, compared to 56% at 10 years. A poorer PCC score was the most sensitive marker for the 44% below ‘peer level’ at 10-year-of-age. The best predictor of ‘peer level’ speech proficiency at 10 years was speech proficiency at 5 years. A high number of ST-visits received did not improve the probability of achieving ‘peer level’ speech, and many children seemed to have received excessive amounts of ST-visits without substantial improvement.

**Conclusions:**

It is important to strive for speech at ‘peer level’ before age 5. Criteria for speech therapy intervention and for methods used needs to be evidence-based.

## Introduction

Treatment outcomes after primary palatal surgery in patients born with cleft lip and palate (CP ± L) have been extensively studied.^1,2^ Still the evidence for best surgical treatment is weak.^3^ Mostly, outcomes have been studied in relation to surgical technique and timing of surgery.^2,4,5^ The Scandcleft project, a randomized controlled trial, commenced in 1997 involving ten cleft centers in five countries (Denmark, Finland, Norway, Sweden, UK) to find evidence for the best surgical practice in children (n = 448) born with unilateral cleft lip and palate (UCLP).^6^ The Scandcleft prospective multi-center project included three parallel trials (Trial 1, Trial 2 and Trial 3).^6^ In each trial, one of the local surgical protocols (Arm B, Long delay in hard palate closure; Arm C, Simultaneous hard and soft palate closure; Arm D, Early hard palate closure with vomer flap) was tested against a common protocol (Arm A, Short delay in hard palate). Two languages were included in each trial; Trial 1 Danish and Swedish, Trial 2 Finnish and Swedish, Trial 3 English and Norwegian, therefore restricted speech material developed for cross-linguistic assessment was used.^7^

Regarding speech outcome, there is so far no strong evidence from the Scandcleft trials to recommend any specific surgical procedure.^8^ Whilst speech outcome at age 5 and 10 respectively, revealed few differences across surgical procedures,^8–10^ differences across centers using the same surgical protocol were found.^11–13^ Thus, familiarity with the primary surgery and operator skill seemed to outweigh the importance of the protocol.^14^ This was in part supported by Slator and colleagues,^5^ who reported higher odds of a good articulation score in children (n = 211) operated by surgeons with higher volumes but also that children receiving later surgery of the soft palate and the lip were more likely to have reduced intelligibility at age 5. Other factors, such as secondary pharyngeal surgery, hearing impairment, and speech therapy intervention might have an impact on both burden of care for the child, but also on speech outcome in a longitudinal perspective.^15,16^ Therefore, this longitudinal study using the datasets of speech with 5- and 10- year-olds participating in the Scandcleft project, focuses on the longitudinal development of speech in relation to secondary pharyngeal surgery and speech therapy intervention. Hearing data was not sufficient for a longitudinal follow-up.

### Longitudinal Perspective

It is challenging to collect and evaluate long-term data. To our knowledge, there are six published studies with longitudinal results regarding velopharyngeal function (VP-function) and articulation at ages 4–5 and at 10 years, comprising in total approximately 300 children with UCLP.^4,17–21^ In these studies, different primary surgical protocols had been followed and the focus of the studies varied. For example, Karnell & Van Demark^17^ investigated the impact of articulation score and VP-closure at age 4 years on articulation score at age 10. Lohmander and colleagues^19^ investigated the influence of age at hard palate repair (3–8 years) after early soft palate repair (6 months) on speech. The relationship between speech and communicative attitude at 10 years of age was the focus of the study by Havstam et al.^20^ No information on secondary pharyngeal surgery and speech therapy intervention was included in the study by Havstam et al.,^20^ whereas the other five studies had data on secondary pharyngeal surgery but limited information on speech therapy. In summary, the proportion of velopharyngeal competence (VPC) varied between 40% and 73% (mean 55%) at age 4–5 years and between 62% and 94% (mean 82%) at age 10. Between 10% and 43% of the children had received secondary pharyngeal surgery at age 10. Articulation proficiency based on assessed error variables, varied between 40% and 60% (mean 53%) at age 4–5 years and between 80% and 100% (mean 90%) at age 10. The limited information on speech therapy intervention revealed that 23%,^19,21^ and at least 61%^4^ had received intervention. No information on amount or type of speech therapy was included. Lack of information on hearing was reported in all six studies.

### Secondary Pharyngeal Surgery

Competent velopharyngeal function is important for speech development. Therefore, secondary pharyngeal surgery is performed in children with velopharyngeal incompetence (VPI), with the intention to reduce passive cleft speech symptoms (hypernasality, nasal air leakage and weak pressure on consonants) and to give optimal conditions for oral speech sound production. Rate of secondary pharyngeal surgery has sometimes been reported as an outcome measure.^22^ However, this measure is difficult to compare between different centers, as the thresholds for secondary pharyngeal surgery can vary substantially depending on, for example, the attitude of both the staff and the families as well as access to speech analysis and visualizing methods of the VP-port.^23^ Among 5-year-old children born with CP ± L included in a large-scale national audit in UK (n = 1110), 36% had structurally related problems likely to be related to VPI or symptomatic fistulas.^1^ Of these, about 10% had received secondary pharyngeal surgery eliminating the passive VP-symptoms, 10% had secondary pharyngeal surgery without eliminating the VPI and about 17% had not undergone secondary surgery. In a later study of 5-year-olds with CP ± L, based on the database CRANE (n = 3157) in the UK, around 30% had structurally related problems with impact on VP-function.^24^

The majority of outcome studies after secondary pharyngeal surgery are retrospective studies based on chart reviews.^25,26^ A few small-scale studies have used good clinical practice with speech assessments based on pre- and postoperative audio or video recordings presented in a randomized order for blinded speech-language pathologists assessing the speech.^27–30^ In these studies, the outcome of different surgical methods such as sphincter pharyngoplasty (n = 20),^27^ autogenous posterior pharyngeal wall augmentation (n = 14)^28^ and pharyngeal flap surgery (n = 23; n = 21)^29,30^ was investigated. They all reported an improvement of VP-function without reaching normalized VP-function in most cases.

### Speech Therapy Intervention

It has been estimated that approximately 50% of children with repaired cleft palate will require speech therapy.^31^ Unfortunately, studies seldom report any descriptive data on the type of speech therapy due to lack of consistent record keeping or different levels of detail in documentation or no documentation at all, for example, at local hospitals or schools.

Commonly, studies have only reported if the children have received speech therapy or not,^32^ but frequency, dosage, and type of intervention have rarely been reported. Sell and colleagues,^33^ reported number and duration of speech therapy visits but the impact of speech therapy on speech outcome was not reported.^33^ Whilst uncertain and little evidence of the effectiveness of any specific speech intervention based on statistical analyses on group aggregated data has been reported,^34^ a recent meta-analysis of individual data from available published studies showed an 80% probability for children born with cleft palate to improve speech during speech therapy.^35^ However, the probability of their speech reaching the level of their peers without a cleft was much lower (10–34%) depending on age.^35^ No study has included the effect of speech therapy in addition to surgical repair in the evaluation of primary treatment for cleft palate.

As differences in speech outcomes in children born with UCLP cannot be fully explained by the primary surgical protocol used, it is important to also study the speech development in a longitudinal perspective and include the impact of secondary pharyngeal surgery and speech therapy in the speech outcome. The Scandcleft project comprises a large number of participants with similar procedures, documentation and analysis of their speech. Therefore, the material should provide sufficient quality for this purpose. Thus, the aim of the present study was to investigate the speech development longitudinally between 5 and 10 years of age, in children with repaired UCLP, using the Scandcleft dataset. The following questions were posed:

1. What was the overall development of the children's speech proficiency (articulation and VP-function) from 5 to 10 years of age?
2. How can the speech of children whose speech proficiency is not at ‘peer level’ at 10 years be characterized? What was their speech like at 5 years of age?
3. How does the number of speech therapy visits between 5 and 10 years of age relate to the child's speech proficiency at 10 years, and what factors are predictive of whether a child's speech proficiency at 10 years is at ‘peer level’ or not?

## Methods

### Data Source

Data was extracted from the Scandcleft 5- and 10-year speech datasets.^8–10^ This analysis was based on phonetic transcriptions of standardized video recordings of the Scandcleft single word test.^7^ Two speech-language pathologists (SLPs) with the same native language as the child and experienced in cleft palate speech had transcribed the target sounds in the test by narrow phonetic transcription according to the IPA and the extended IPA for disordered speech.^36^ The protocol of the project included the reported number of speech therapy visits, both at the cleft center and at locally based SLP services, and all secondary pharyngeal surgeries performed. Pure tone audiometry was part of the protocol but was excluded from this study due to a large number of missing tests.

In the sample, 241 children did not receive secondary pharyngeal surgery between 5 and 10 years of age, whereas 79 (25%) did. Of these, 73 had a pharyngeal flap and another six had secondary pharyngeal surgery both before age 5 and between 5 and 10 years of age with a different combination of methods. Further, 29 (9%) children had secondary pharyngeal surgery before age 5 (12 had a pharyngeal flap, 11 Furlow palatoplasty, one had a palatoplasty according to Sommerlad, and one had a Hynes pharyngoplasty). Another four children had two secondary procedures before age 5 (2 Furlow palatoplasty and pharyngeal flap, 1 Sommerlad palatoplasty and pharyngeal flap, and 1 pharyngeal flap and a re-repair of the flap). Thus, 108 (34%) children had received secondary pharyngeal surgery at age 10; 29 before age 5 and 79 between 5 and 10 years of age.

Written informed consent to participate in this study was provided by the participants’ legal guardian. Ethical approval was obtained from the appropriate ethical committee in each country and the study was registered in the ISRCTN (Trial registration: ISRCTN299932826).

### Participants

To be included in the original Scandcleft project children should be of Caucasian origin, have a non-syndromic complete UCLP or a soft tissue bridge not more than 5 mm. Further, the primary language in the country of origin should be spoken at home.^6^ In the present longitudinal study the participants had to have a complete dataset from both their 5- and 10-year assessments according to the Scandcleft procedure. From the original 448 children randomized to the trials, 320 children fulfilled the inclusion criteria for the present study: complete datasets from the two included time points. One British center (n = 17) did not continue the follow-up after 5 years of age, children from this center are therefore excluded. Incomplete datasets were due to, a failure to attend, a technical fault or lack of information on the number of ST-visits. Thus, from Arm A 162, Arm B 68, Arm C 34, and Arm D 56 children participated. From Center 1–9 were 77, 37, 22, 38, 2, 31, 50, 28, and 35 children.

### Speech Material

Restricted speech material, including similar speech sounds for each language had been developed to rule out, as much as possible, the impact of five different languages. The included consonants were phonetically similar, had the same position in the words and a similar phonetic context; no other pressure consonant other than the target sound if possible, no nasal consonants in words with an oral target consonant and no consonant clusters.^7^ All consonants /p b t d k g f v s n/ were in stressed initial position three times, except for /s/ which had an additional three in final position. Thus, the speech material included 33 target sounds in 33 words. The exception was Finnish. As /b d g f/ are not included in their language, the speech material included six words for each of the target consonants /p t k/, and no words with /f/. Therefore, there were only 30 target sounds in 30 words in the Finnish word list.^7,10^

### Speech Proficiency

#### Velopharyngeal Composite Score, VPC-Sum

Velopharyngeal function was assessed with the composite score VPC-Sum.^37^ This score was based on three variables: hypernasality, passive VP-symptoms and non-oral errors. To rule out the language impact as much as possible, the first nine words in the single word test were edited to a string without pauses. These words had a similar phonetic context across languages and the vowels were high or semi-high as these are thought to be the most vulnerable for hypernasality.^38^ This string was rated by three SLPs in a two-step procedure, two SLPs with the same native language as the child, and one SLP with another native language. First, the SLPs decided if the resonance was within normal limits or not. If not, it was rated as mild, moderate, or severe hypernasality. The outcome was decided by a majority decision across three raters. The ratings were given a new VPC-Sum score; normal resonance (0) = 0, mild hypernasality (1) = 1, moderate-severe hypernasality (2–3) = 2. Number of VP-symptoms (audible nasal air leakage accompanying consonants, reduced pressure on oral pressure consonants, nasal consonants for voiced plosives) and non-oral errors (pharyngeal and glottal fricatives and stops, nasal for unvoiced stop or fricative and active nasal fricatives) were calculated from the phonetic transcriptions. A new VPC-Sum score was given for each one of the variables, VP-symptoms, and non-oral errors; 0–2 affected consonants = 0, 3–5 affected consonants = 1, and 6 or more affected consonants = 2. Thus, a VPC-Sum composite score based on hypernasality, VP-symptoms and non-oral errors could vary between 0–6. In this study a dichotomized VPC-Sum score was chosen; 0–1 represents a competent VP-function (‘peer level’) and 2–6 an incompetent VP-function.^37^

#### Percentage of Consonants Correct, PCC

Consonant proficiency was measured as the percentage of consonants correct (PCC).^39^ This method was originally developed on connected speech as a severity measure by Shriberg & Kwiatkowsky^40^ but later modified and used on target sounds in single words.^41,42^ Each target consonant was scored as correct or incorrect based on the phonetic transcription. Any deviance of place and manner of articulation was scored as incorrect. If a target consonant was produced correctly except for passive VPI-symptoms (nasal air leakage or reduced pressure) it was scored as correct. Whilst other distortions were not considered correct, the PCC measure in this situation was considered to be the original.^39^ Besides the continuous measure of PCC between 1 and 100 we reported a dichotomized PCC being at ‘peer level’ (≥91%, corresponding to 0–3 errors) or not. This corresponds approximately to PCC-scores in typically developing 5-year-old children in United Kingdom (90.4%) and Sweden (91%).^43,44^ This level was earlier used in the Scandcleft trials^11–13^ and corresponds to the 16^th^ percentile or −1 SD from the mean in a normal distribution.

#### Speech Errors

Speech errors were categorized as active cleft speech characteristics (CSC) or developmental speech characteristics (DSC).^10^ The CSCs were further divided into oral errors (retracted articulation of anterior consonants to palatal, velar or uvular place of articulation) and non-oral errors (glottal, pharyngeal, nasal for unvoiced plosive, active nasal fricative). Developmental speech characteristics included errors typically seen in younger children such as stopping and fronting but also interchangeable use of fricatives. Both CSCs and DSCs were reported in categories; 0 errors = 0, 1–2 errors = 1, 3 or more errors = 2.

### Speech Therapy Visits

Number of speech therapy visits (ST-visits) between age 5 and 10 years were collected on a specific document for each participant by the SLP at the cleft center. The SLP also sent a questionnaire to local hospitals or other services of speech intervention. All types of visits were included: routine visits, follow-ups, and speech intervention. We had no information on the frequency of visits or the focus of speech intervention. The participants were divided into four groups depending on number of ST-visits and clinical experience of what constitutes “fewer,” “typical,” and “more” visits. 1) Those receiving six or fewer ST-visits (*n *= 163), 2) those receiving between seven and 28 visits (*n* = 79), 3) those receiving between 29 and 99 visits (*n* = 65), and 4) those receiving 100 or more visits (*n* = 16). The first group had only routine follow-ups once a year. The second group received a rather common number of routine follow-ups and speech intervention. The third group had a substantial number of speech interventions, and the fourth group had an unusually high number of ST-visits.

### Statistical Analysis

To compare each child's speech proficiency at 5 and 10 years we used both the continuous PCC-scores and the dichotomized VPC-Sum score. Children were categorized as being at ‘peer level’ if their scores were above the specified threshold for PCC (≥91%) and VPC-Sum (0–1). This was done by creating, and visually inspecting, a “Brinley plot” (scatterplot) for the PCC- scores at 5 and 10 years of age and a 2 × 2 contingency table regarding whether PCC and VPC-Sum were at ‘peer level’ or not.

Speech outcome profiles for the children with PCC and/or VPC-scores below ‘peer level’ at age 10 were visualized in a heatmap including the variables VPC-Sum, oral errors, non-oral errors, and DSCs. The participants were roughly sorted in accordance with the type and degree of errors they made. These visualizations of types and degrees of errors made at 10 years of age were compared with the same data at 5 years of age.

To investigate how the number of ST-visits attended between 5 and 10 years of age were related to the probability that PCC at 10 years of age was at ‘peer level’ or not, only children who were not at peer-level at 5 years of age were included. This was because most of the children at ‘peer level’ were already at ceiling performance and therefore they would skew the modeling. First, we visually graphed the relationship between the different variables in scatterplots. To formally analyze the relationship, we also used a logistic regression model in which whether speech proficiency at 10 years of age was at ‘peer level’, or not, was the outcome. The model included the following predictors: (1) PCC score (measured continuously) at 5 years of age, (2) VPC-Sum at 5 years of age (could vary between 0–6, as described above), (3) the number of ST sessions the children attended between 5 and 10 years of age, and (4) whether the child received secondary VP-surgery, or not, between 5 and 10 years of age [dummy coded as 0 = no VP-surgery, 1 = had received VP-surgery]. To begin with, we also entered predictors (5) what Center the children attended and (6) what Surgery-arm the children had received as separate group predictors with random intercepts in a generalized mixed-effects logistic regression model. However, the random effects variance was estimated to be very small in the mixed-effects model and neither centers, nor Surgery-arms differed enough to produce different intercepts in the model. We therefore report on a model that neither included centers nor surgery-arms as random effects, but instead used a more parsimonious, traditional logistic regression model with only fixed effects.

The regression coefficients in a logistic model describes the probability of the outcome based on the predictors on a log-odds scale, or in “logits.” To describe the logistic regression result in understandable terms, we back-transformed the logit output to the probability scale and illustrate the predicted probability of a child's speech to be at ‘peer level’ based on the various predictors.

All statistical analyses were carried out in *R,*^45^ and the generalized mixed-effect logistic regression model was fitted using the *glmer-*function in the *lme4* package.^46^

## Results

### Comparing Speech Proficiency at 5 and 10 Years

**Figure 1.**
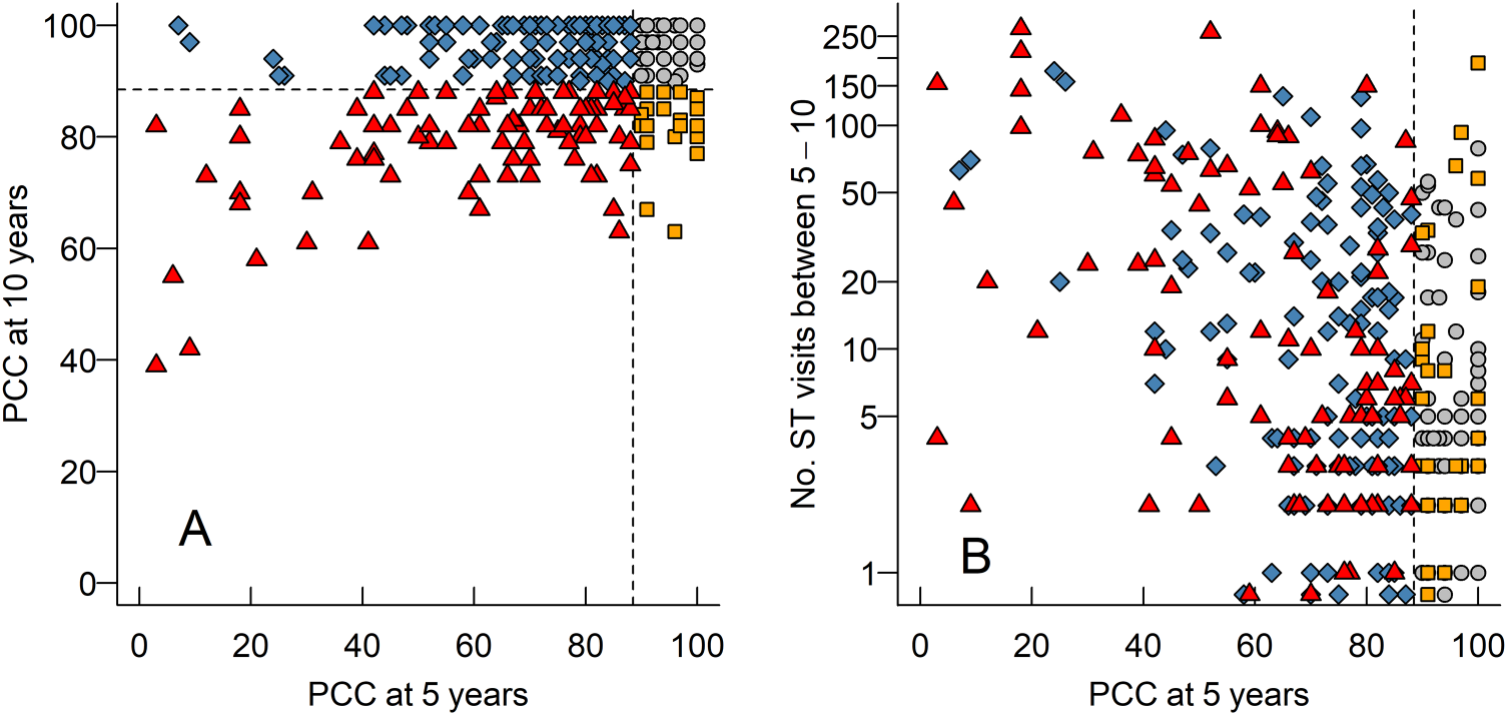
(A) A scatterplot (“Brinley plot”) of each child's percentage consonants correct score (PCC) (marked as different symbols) at 5 years of age (x-axis) and 10 (y-axis). In all panels, dashed horizontal and vertical lines illustrate our cut offs for considering PCC to be at ‘peer level’ or not. Triangles – PCC were not at ‘peer level’ at 5 or 10 years of age, diamonds - PCC were below ‘peer level’ at 5 years of age but at ‘peer level’ at 10-years of age, circles - PCC were at ‘peer level’ at both time points, and squares - PCC were at ‘peer level’ at 5 years of age, but not at 10 years of age. (B) A scatterplot of each child's PCC score at 5 years of age and their number of ST-visits between 5 and 10 years of age. Note that the y-axis is logarithmic.

More children had a speech proficiency, assessed as PCC and VPC-Sum, at ‘peer level’ at 10 years of age (*n* = 180) than at 5 years of age (*n* = 75) (Table 1). In other words, many children improved their speech between 5 and 10 years of age. In Figure 1A each child's PCC-score can be followed from age 5 to 10 years. Of the 214 children assessed as *not* being at ‘peer level’ on PCC at 5 years of age, 125 (58% of those 214) had caught up and were on ‘peer level’ at 10 years of age. A group of 26 children was deemed to be at ‘peer level’ on PCC at 5 years of age, but not at 10 years of age (marked as squares in Figure 1A). Many of the children with the poorest PCC-scores at 5 years of age, also had the poorest scores at the 10-year measurement (Figure 1A). Regarding VPC-Sum of the 147 children assessed as not being at ‘peer level’ on VPC-Sum at 5 years of age, 101 (69% of those 147) had caught up and were at ‘peer level’ at 10 years of age. A group of 21 children were at ‘peer level’ on VPC-Sum at age 5 but not at age 10 (Table 1).

**Table 1.** A Contingency Table for the Number of Children Whose PCC-Scores and/or VPC-Sums Were at ‘Peer Level’ (PL) (Above our Cutoff of 91% for PCC and Score 0–1 for VPC-Sum), or not, at 5 and 10 Years of age.

Speech proficiency at 5-year	Speech proficiency at 10-year				Row sums
	PCC PL VPC PL	PCC PL VPC not PL	PCC not PL VPC PL	PCC not PL VPC not PL	
PCC PL VPC PL	50	7	17	1	75
PCC PL VPC not PL	17	6	2	6	31
PCC not PL VPC PL	57	5	28	8	98
PCC not PL VPC not PL	56	7	26	27	116
Column sums	180	25	73	42	*N* = 320

*Note.* The median PCC score at 5-years was 82% (min-max: 3–100) and at 10-years was 94% (39–100). The median VPC-Sum at 5-years was 1 (on a scale from 0–6; min-max: 0–6) and at 10-years was 0 (0–6).

### Speech Outcome Profiles for the Children not at ‘Peer Level’ Speech at 10 Years of age

**Figure 2.**
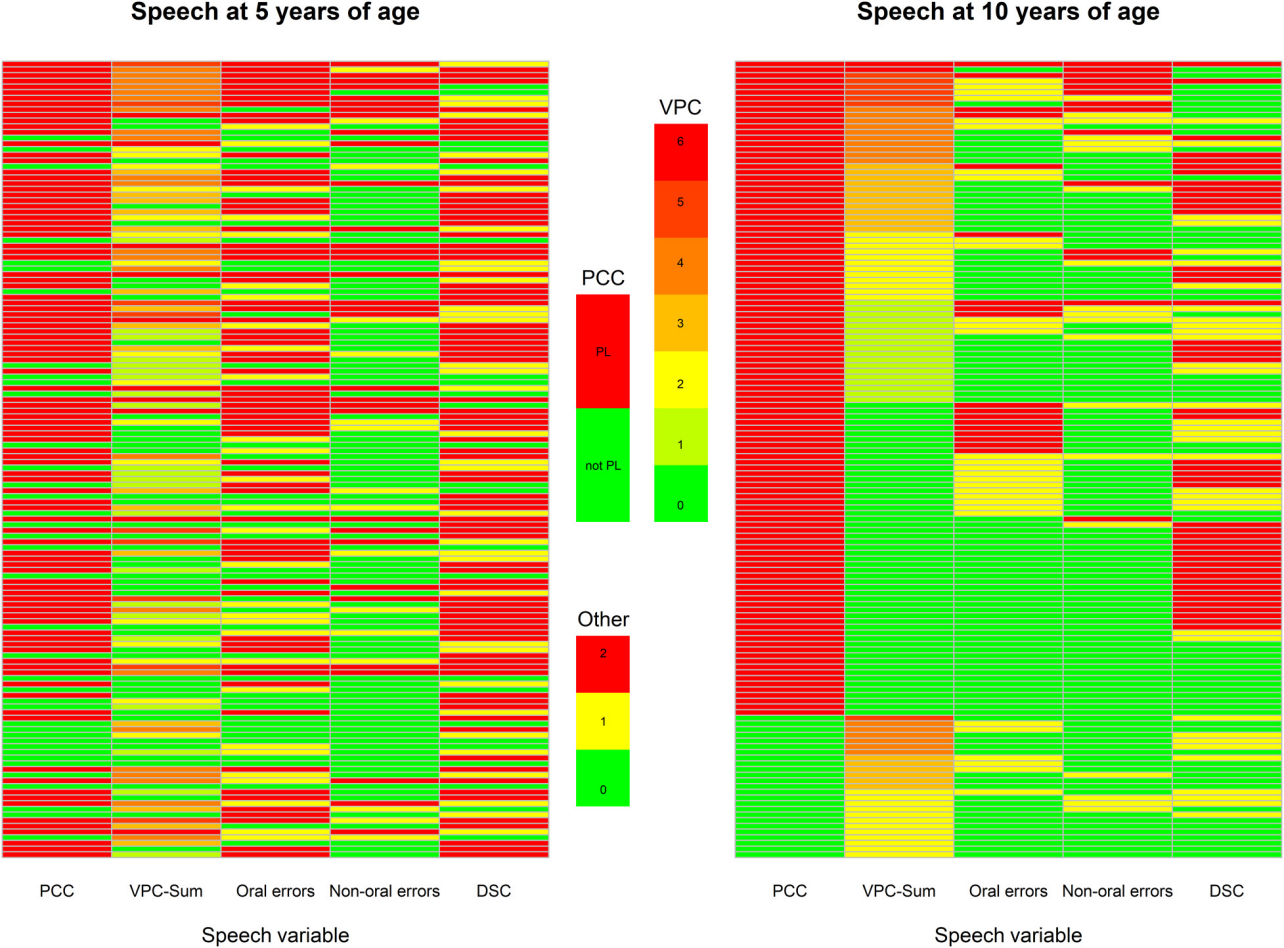
Two heatmaps of the 140 children whose speech proficiency (PCC and/or VPC-Sum) were not at ‘peer level’ at 10-years of age. Each row is one child and each column one colour coded speech variable. To the left is a heatmap of the children's speech on the five variables at 5 years of age. To the right, the corresponding heatmap for the children at 10 years of age. PCC is coded as at ‘peer level’ (PL = PCC > 91%) in green or not (not PL = PCC < 91%) in red. VPC-Sum was illustrated from green (competent = 0) to red (incompetent = 6), and the speech errors (oral errors, non-oral errors and DSC); green (no errors = 0), yellow (1–2 errors) and red (3 or more errors). The outcomes were ordered according to their speech proficiency at 10-years of age. The same ordering was kept when illustrating their speech at 5-years of age (left panel). Note. VPC-Sum = Velopharyngeal Competence-Sum. DSC = Developmental Speech Characteristics.

The speech profiles of the 140 children whose speech was *not* at ‘peer-level’ at 10 years of age are presented in two heatmaps (Figure 2). These children (rows) were ordered according to their speech proficiency at 10 years of age (Figure 2, right panel) as this was our grouping variable. The same ordering of the children was kept to illustrate their speech at 5 years of age (Figure 2, left panel) so that changes between the panels for individual children could be tracked. The reason for not reaching ‘peer level’ was in most children a low PCC score, for about one third in combination with VPI. Only a minority had just VPI. Non-oral errors were mainly related to VPI, and oral errors and DSC were seen irrespective of a competent VP-function or not.

### Predicting Speech Proficiency at 10 Years of age

In Table 2, we list how the four groups of children illustrated in Figure 1A (below peer at both ages; ´peer leveĺ only at 10-years of age; ‘peer level’ at both ages; ‘peer level’ only at 5 years of age) were differentiated with regards to the number of ST-visits they had received prior to 5 years of age (median 10, min-max 2–176) and between 5 and 10 years of age (median 6, min-max 0–271). The highest number of ST-visits, both before 5 years of age and between 5–10 years of age, was found in the groups below ‘peer level’ at both ages and ‘peer level’ only at 10 years of age. Further, we list the number of Secondary pharyngeal surgery the four groups of children had before 5 years of age (*n* = 29; 9%) and between 5 and 10 years of age (*n* = 79; 25%). Figure 1B illustrates the relationship between PCC at 5 years of age and the number of ST-visits the children made. Note that the *y*-axis is logarithmic because of the large dispersion in the number of ST-visits the children received. All predictors for 10-year speech proficiency (PCC at 5 years, VPC-Sum at 5 years, Number of ST-visits between 5 and 10 years, Secondary pharyngeal surgery between 5–10 years) were quite strongly correlated (absolute *r*s between .13 and .50). There was, as is reasonable, a *selection bias* such that children with poorer PCC- scores at 5 years of age *on average* attended more ST-visits. However, even some children whose speech at 5 years of age was at ‘peer level’ attended many ST-visits. Note that in the analysis on predicting speech proficiency at 10 years of age, we only include those children not already at peer-level speech at 5 years of age (*n* = 214).

**Table 2.** Cleft Care for the Four Groups of Children Illustrated in Figure 1: those with PCC below peers at both ages (red triangles in Figure 1), those at peer-level (PL) only at 10-years (diamonds), those at PL at both ages (circles), and those at PL only at 5-years (squares).

Variable	Below peers at both ages *n* = 89	PL only at 10 years *n* = 125	PL at both ages *n* = 80	PL only at 5 years *n* = 26	Total *N* = 320
Number of children receiving SPS before 5 years (%)	6 (7%)	16 (13%)	5 (6%)	2 (8%)	29 (9%)
Number of ST-visits before 5 years, median (min – max)	15 (3–176)	13 (2–105)	7 (3–57)	6 (3–67)	10 (2–176)
Number of children receiving SPS between 5 and 10 years (%)	30 (34%)	26 (21%)	18 (22%)	5 (19%)	79 (25%)
Number of ST-visits between 5 and 10 years, median (min – max)	10 (0–271)	12 (0–175)	4 (0–79)	6 (0–190)	6 (0–271)

*Note.* Secondary pharyngeal surgery (SPS) indicates that at least one secondary pharyngeal surgery had occurred. Nine children had more than one SPS.

**Figure 3.**
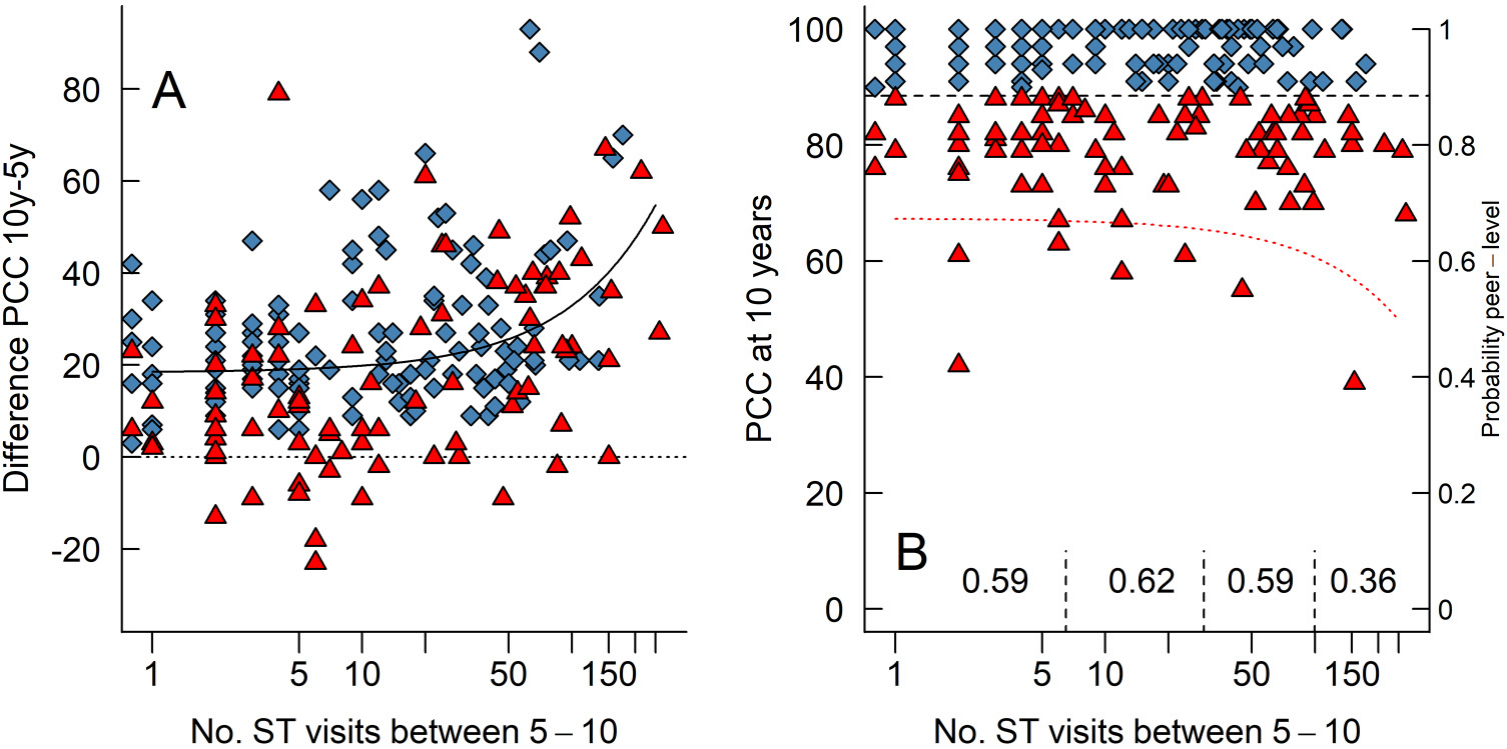
(A) A scatterplot of each child's number of ST-visits between 5 and 10 years of age and their difference in PCC-scores between those ages (calculated as PCC 10y minus PCC 5y, so that positive values indicate improvement). The dotted horizontal line indicates zero improvement. The black “curve” is the linear relationship between the x- and y-axis; note that a linear relationship looks like a curve when one of the axes are logarithmic. (B) A scatterplot of each child's number of ST-visits between 5 and 10 years of age and their PCC-scores at 10 years of age. The dotted curve is the result of the logistic regression and is plotted against the right-hand y-axis that indicates the predicted probability that a child's speech is at ‘peer level’ or not. The numbers written on the bottom are the proportion of children with ‘peer level’ speech in each, demarcated by the vertical dashed line.

Figure 3A illustrates the relationship between the number of ST-visits the children made and their improvement in PCC-scores (calculated as PCC at 10 years of age minus PCC at 5 years of age). As can be seen, the children who attended more ST-visits made greater numerical improvements in their PCC-scores. However, this relationship can largely be explained by the stated *selection bias* and a *ceiling effect* such that children with poorer PCC-scores at 5 years of age both have more room to improve and attended more ST-visits.

Figure 3B illustrate the relationship between the number of ST-visits the children received and their PCC-scores at 10 years of age. Note that with more ST-visits, a smaller proportion of children had ‘peer level’ consonant proficiency. More specifically, after around 50 ST-visits the probability to reach ‘peer level’ did not increase. When we group the children into those receiving six or fewer ST-visits (*n *= 91), those receiving between seven and 29 visits (*n* = 60), those receiving between 30 and 99 visits (*n* = 49), and those receiving 100 or more visits (*n* = 14) then 59%, 62%, 59%, and 36% of those children had speech proficiency at ‘peer level’ at 10 years of age, respectively. Thus, children who received more ST-visits numerically improved more at their PCC-scores (Figure 3A) but were less likely to have ‘peer level’ speech at 10 years of age (Figure 3B).

This impression was confirmed by our logistic regression model that included several predictors. The model overall fit the data to a reasonable degree and correctly sorted 65% of the individuals. Table 3 lists the model coefficients in logit-terms. The only statistically significant predictor was that better PCC-scores at 5 years of age was positively related to having ‘peer level’ speech at 10 years of age. To understand the logit coefficients, we can back-transform the logit output to the probability scale. If we set the predictors number of ST-visits and VPC-Sum at 5 years of age to their respective medians for the 214 children included in the analysis and the predictor secondary pharyngeal surgery to none received between 5 and 10 years of age, then a child who has a PCC score of 40 at 5 years of age has a predicted probability of reaching ‘peer level’ at 10 years of age of 47%. A similar child who has a PCC score of 80 at 5 years of age has a predicted probability of reaching ‘peer level’ at 10 years of age of 70%.^1^

**Table 3.** Coefficients in the Logistic Regression Expressed in Logit.

Predictor	Logit	LL	UL	*p*	VIF
Intercept	−1.186	−2.708	0.260	.12	-
PCC at 5 years (continuous measure)	0.025	0.007	0.043	.007	1.4
VPC-Sum at 5 years	0.055	−0.126	0.241	.55	1.6
No ST-visits between 5 and 10 years	−0.003	−0.010	0.004	.43	1.2
Secondary pharyngeal surgery between 5–10 years	-0.546	-1.291	0.190	.15	1.3

*Note*. *LL* and *UL* = Lower and Upper Limit of 95% confidence intervals are the estimates. *VIF* = Variance inflation factors.

Most importantly for our question was that the number of ST-visits received was not positively related to the probability of the speech being at ‘peer level.’ The dotted curve in Figure 3B illustrates the relationship between number of ST-visits and the (back-transformed predicted) probability to have ‘peer level’ speech (tracked on the right-side *y*-axis) when the predictors PCC and VPC-Sum at 5 years of age are set to their respective medians and when the predictor secondary pharyngeal surgery is set to none received between 5 and 10 years of age. As can be seen, our model follows the raw data well (compare the curve to the proportions written at the bottom of the figure), such that with more ST-visits, the probability to reach ‘peer level’ does not increase. There were no problems with multicollinearity in the model as illustrated by the low variance inflation factors (VIF) values in Table 3.

## Discussion

This study aimed to investigate speech development longitudinally from age 5 to 10 years, including the impact of secondary pharyngeal surgery and number of ST-visits in 320 children born with unilateral cleft lip and palate. Many children whose speech proficiency was below ‘peer level’ at 5 years of age improved, but only 56% were at ‘peer level’ at age 10. Thus, 44% (n = 140) were below ‘peer level’ at 10 years of age. A few of these had only VPC-Sum below ‘peer level,’ but more common were PCC alone or in combination with VPC-Sum below ‘peer level.’ Generally, even the group below ‘peer level’ at age 10 had fewer speech errors at age 10 compared with age 5 but notably, developmental speech characteristics (DSC) more commonly remained at age 10 compared with the cleft speech characteristics (CSC) oral and non-oral errors. In fact, one third (34%) of the 10-year-olds below ‘peer level’ had DSCs. The number of ST-visits the children attended was positively correlated to a numerical improvement in PCC-scores (Figure 3A), but this *numerical result* could largely be explained by the fact that the children with the poorest speech at age 5 years *generally* received more ST-visits and had more room to improve on their speech measures. More to the point, many children seemed to receive excessive ST care without substantial improvements in the probability of achieving ‘peer level’ speech (Figure 3B). Further, the probability to achieve ‘peer level’ speech at 10 years of age was highly related to better PCC-scores at age 5, whilst VPC-Sum and secondary pharyngeal surgery had a very small impact (Table 3).

Although, most children improved their speech proficiency, a group of 26 children were deemed to be at ‘peer level’ at age 5 but not at age 10. A closer look into their speech outcome at age 10 revealed that the lower PCC-score was most often related to distortions of s-articulation. PCC was based on phonetic transcription with the same assessment methodology and the same speech material at both ages. However, the technical quality of the recordings had improved during these years, which might have improved the possibility to perceive s-distortions. It might also be that the SLPs unconsciously had higher expectations of the pronunciation at age 10, as more children in the general population have more s-distortions at age 5 (28%) compared with age 10 (15%).^44^ Further, some of the SLPs performing the transcription at age 10 were not part of the 5-year assessment and for those who took part in both transcriptions several years had passed. All these scenarios could have had an impact on the listeners perception.^47–49^ Another possibility is a change in dentition and occlusion among the participating children between age 5 and 10, causing more difficulties with the sibilant fricative /s/^50^ as poor maxillary growth has been related to anterior oral errors.^51^ The high number of DSCs (Figure 2), in the group not at ‘peer level’ at age 10, was also highly related to anterior errors of /s/, and less commonly also to bilabial articulation of labiodental fricatives (/f/), errors of voicing, fronting and stopping. There are few studies of DSCs or phonological processes in children with CP ± L, and even more rare in children after 5 years of age. In two recent studies 4–7-year-old children with CP ± L had more phonological errors than CSCs^16^ and among 9–29-year-old individuals with CP ± L or submucous clefts, 15% still had phonological processes.^52^ Thus, this is an area that needs to be investigated longitudinally. The remaining CSCs of non-oral errors were in most cases related to a VPC-Sum not at ‘peer level’ at age 10, that is an inability to close the port between the oral and nasal cavity, and in a few cases, they seemed to remain from an earlier incompetent VP-function at age 5. Warren^53^ hypothesized that these types of compensatory strategies are manifestations of regulation and control in order to maintain speech pressure in a speech regulating system. Therefore, the place of articulation is most often moved to a place behind the defect,^54,55^ in this case the velopharynx.

In this study, number of speech therapy visits (ST-visits) was used as a measure for speech therapy intervention dosage. The variation in the number of ST-visits varied considerably as half of the children had received six or fewer visits, whereas some had received more than 100 visits. Further, a high variation in number of ST-visits was seen among all the four subgroups (Table 2) that is, PCC below peer-level (PL) at both ages; PL only at age 10; PL at both ages; PL only age 5. The highest median number of ST-visits was found in the subgroups ‘below PL at both ages,’ and ‘PL only at age 10’, but surprisingly some children in all four groups had a high number of ST-visits. Around 60% of those receiving less than 100 ST-visits had a consonant proficiency at ‘peer level’ at age 10, compared with 39% for those receiving more than 100 ST-visits (Figure 3B). Thus, the group of children receiving most ST-visits were less likely to have peer level PCC at age 10. As this is not an experimental trial, there is of course, as mentioned, a selection bias such that children with poorer speech at 5 years of age (and thus having poorer prospects at 10 years of age) received, on average, more ST visits which can partly explain the lack of an effect of the number of ST visits. However, it is clear that there can be excessive amounts of ST visits given without having any effect. The probability of reaching ‘peer level’ speech did not increase after around 50 ST-visits (Figure 3B). If no or limited improvement is seen, clinicians should evaluate the reasons for the child's speech difficulties, intervention method used, and indication for intervention. So, even if many children improved their consonant proficiency they did not reach ‘peer level’, which is in line with the meta-analysis of individual improvement after speech therapy intervention.^35^ However, Sand and colleagues highlighted that many of the included studies were not designed to reach ‘peer level’, as they often had set the number of speech therapy visits before therapy started.^35^ In the Scandcleft project a number of children had received an excessive number of ST-visits, without reaching ‘peer level’. We can only speculate on the reasons for these high numbers. An increased risk of Childhood Apraxia of Speech (CAS) has been reported for children with CP ± L,^56^ which could be one reason for the remaining difficulties despite many speech therapy visits. It cannot be ruled out that some children with structural defects (fistulas, VPI) had received a high number of speech therapy visits, even though they presumably did not have the structural prerequisite for speech at ‘peer level.’ Another possibility is that parental worry and concern^57^ or pressure from other team members are reasons for ST-visits, rather than clear evidence-based indications. It is therefore important to further develop and evaluate effectiveness of speech therapy intervention for children with CLP. In the Scandcleft project children with genetic syndromes were excluded before randomization into the project. A formal screening of neurodevelopmental disorders (NDD) was not part of the project. However, in recent studies an increased number of overlapping neurodevelopmental co-morbidities (NDDs), for example developmental language disorders (DLD), attention deficit-/hyperactivity disorder (ADHD), Autism spectrum disorders (ASD), Intellectual disability (ID), learning disabilities, and developmental coordination disorder (DCD), have been reported in individuals with non-syndromic orofacial clefts.^58,59^ Further, academic deficits in all ages,^59^ and suggested structural brain differences^60^ between individuals with non-syndromic oral clefts and controls have been reported in earlier studies. There is also emerging evidence for a common origin of the orofacial clefts and the brain development.^61,62^ Therefore, it might be possible that that we have a higher prevalence of NDDs than earlier thought in the present cohort, as well as in other cohorts of children with clefts. This possibly higher prevalence of NDD might be a reason for many ST-visits in children with PCC not at ‘peer level.’ NDD is also suggested as a factor that should be looked at in future studies of the development of speech and language skills in children with CP ± L.

In the present study, poor speech at 5 years of age was the only statistical significant predictor for speech below level of peers at 10 years of age. This is despite the SLPs generally identifying those with the poorest speech at 5 years of age, in the sense that they generally received more ST-visits. Thus, the strongest predictor we can point at is achieving good speech at the age of 5 years, which should be strongly prioritized. While this has long been the goal for cleft centers^31^ up to 50% of children with UCLP are commonly reported to have speech difficulties at age 5 years.^1,4,9–13,21,63^

A rather high proportion of the participants (34%) have had secondary pharyngeal surgery. This figure is lower compared with 43% in a study on 10-year-olds (n = 69) with UCLP^4^ but much higher compared with 9% in children (n = 55) at the same age and with the same cleft type.^21^ Most troublesome was that secondary pharyngeal surgery had a small impact on the probability to reach ‘peer level’ of speech at age 10. Sell et al.,^15^ found that a history of secondary pharyngeal surgery was predictive of poor structural outcome. This together with earlier reports of improved speech without reaching normalized speech,^1,27–30^ urge future studies to have careful analysis of possible factors associated with good and poor outcome after secondary pharyngeal surgery.

In addition to optimal primary palatal repair, early identification of risk factors of poor speech/language development would be helpful for the decision of effective treatment interventions before age 5. Babbling and early speech production in children with CP ± L is rather well documented. Whilst typically developing children without clefts prefer anterior consonant placement in late babbling^64^ a remarkable lack of dental/alveolar consonants in children born with CP ± L has been frequently reported in toddlers born with CP ± L.^65–68^

Since there is a continuity between pre-speech and speech production,^69^ phonetic features of babbling have been thought to be able to explain the nature of misarticulations in later speech.^70^ Consequently, a lack of anterior and specifically dental/alveolar plosives in babbling has been shown in children with articulation errors such as retracted oral articulation at age 3 years.^71^ Furthermore, true consonant production at 12 months has been associated with later consonant production,^72^ a low number of dental/alveolar plosives, and a low number of different oral consonants at 18 months of age has been related to a low percentage of consonants correct at age 3 years.^41,73,74^

Hearing loss related to the commonly present OME in children with CP ± L should be treated.^75,76^ An impact on the early consonant production (development) in children with OME-related hearing loss without cleft palate has been shown.^74^ Hearing sensitivity at 18 and 30 months of age was a factor significantly related to early consonant variables and PCC at 3 years of age. Thus, based on the current knowledge, OME-treatment, hearing intervention, and assessment of babbling and early speech variables such as number of different true consonants and presence of dental/alveolar plosives at 18 months of age are recommended when deciding about efficient early intervention.

The next step should be assessment at age 3 years with decision on secondary pharyngeal surgery and/or speech therapy in order to optimize speech at 5 years of age. A recent meta-analysis revealed that speech therapy of different length, setting and procedure has a high probability of benefitting speech in children born with CP ± L, especially if younger than 6 years of age.^35^

## Implications

Speech outcome in children with CP ± L is complex and the interplay across variables related to the surgery, speech, and dental interventions, but also intrinsic factors such as cleft width, hearing, NDD and other genetic factors, are to date not fully understood. Thus, it is important to continue to find predictors for good speech outcome. This study has pointed to the importance of optimizing speech development before age 5, as high PCC and competent VP-function at age 5 were the most important predictors for speech outcome at age 10. Therefore, the outcome from the primary surgery is of utmost importance. If VPI and articulation problems are evident, secondary VP-surgery and speech intervention should be performed before age 5, if possible. Future studies of secondary VP-surgery should aim to find predictors for good speech outcome. Furthermore, it is important to develop evidence-based indications for speech therapy intervention and to develop and evaluate the outcome. Presumably, some children will have speech problems despite the described efforts, so for the future it will also be important to rely on evidence-based speech therapy intervention. It will also be important to further study the impact of cleft width, hearing impairment, genetic syndromes and neurodevelopmental disorders including language variables, on speech outcome.

## Note

Helene Sogaard has moved to a new institution since completing the research, Center for Communication and Welfare Technology, Region of Southern Denmark.

## Notes

1 This is the complete calculations for the two example children. The median number of ST visits among the 214 children included in the analysis was 10 and the median VPC-Sum score was 2. Thus, for a child with no surgery and a PCC score of 40, the calculation would be: -1.186 + 40 × 0.025 + 2 × 0.055 + 10 × -0.003 + 0 × -0.546 = -0.106 That’s the logit for that child, we can get to the odds through exp(-0.106) = 0.8994246 and the probability through 0.8994246 / (1 + 0.8994246) = 0.4735248 or 47%. For a child with a PCC score of 80, the calculation is: -1.186 + 80 × 0.025 + 2 × 0.055 + 10 × -0.003 + 0 × -0.546 = 0.894 After transforming the logit back to the probability scale, we get 70% (given rounding error).
